# Direct oral anticoagulant versus low molecular weight heparin for the treatment of cancer-associated venous thromboembolism: 2022 updated systematic review and meta-analysis of randomized controlled trials

**DOI:** 10.1186/s13045-022-01289-1

**Published:** 2022-05-21

**Authors:** Corinne Frere, Dominique Farge, Deborah Schrag, Pedro H. Prata, Jean M. Connors

**Affiliations:** 1 INSERM UMRS-1166, Institute of Cardiometabolism and Nutrition, GRC 27 GRECO, Sorbonne Université, 75013 Paris, France; 2 Department of Hematology, Pitié-Salpêtrière Hospital, Assistance Publique-Hôpitaux de Paris, 47-83 Boulevard de l’Hôpital, 75013 Paris, France; 3 Department of Medicine, McGill University, Montreal, QC Canada; 4 Research Institute of the McGill University Health Centre, McGill University, Montreal, QC Canada; 5 Department of Internal Medicine (UF 04) : CRMR MATHEC, Maladies Auto-Immunes Et Thérapie Cellulaire, Saint-Louis Hospital, Assistance Publique-Hôpitaux de Paris, 75010 Paris, France; 6 Institut Universitaire d’Hématologie, Université de Paris, EA 3518, 75010 Paris, France; 7 Memorial Sloan Kettering Cancer Center, New York, NY USA; 8 INSERM U944/CNRS, UMR7212, Institut de Recherche Saint-Louis, Université de Paris, 75010 Paris, France; 9 Hematology-Transplantation Department, Saint-Louis Hospital, Assistance Publique-Hôpitaux de Paris, 75010 Paris, France; 10 Department of Medical Imaging, Hematology, and Oncology, Ribeirão Preto Medical School, University of Sao Paulo, São Paulo, Brazil; 11 Department of Medicine, Brigham and Women’s Hospital, Harvard Medical School, Boston, MA USA

**Keywords:** Cancer, Venous thromboembolism, Direct oral anticoagulant, Low-molecular-weight heparin

## Abstract

International clinical practice guidelines have progressively endorsed direct oral anticoagulants (DOACs) as an alternative to low-molecular-weight heparins (LMWHs) monotherapy for the initial and long-term treatment of cancer-associated thrombosis (CAT). Several new randomized controlled trials (RCTs) have recently reported additional results on the safety and efficacy of DOACs in this setting. We performed an updated meta-analysis of all publicly available data from RCTs comparing DOACs with LMWHs for the treatment of CAT. Six RCTs enrolling 3690 patients with CAT were included. Compared with LMWHs, DOACs significantly decreased the risk of CAT recurrence (RR, 0.67; 95%CI, 0.52–0.85), with a non-significant increase in the risk of major bleeding (RR, 1.17; 95%CI, 0.82–1.67), a significant increase in the risk of clinically relevant nonmajor bleeding (RR 1.66; 95%CI, 1.31–2.09) and no difference in all-cause mortality rates. These results increase the level of certainty of available evidence supporting the use of DOACs as an effective and safe option for the treatment of CAT in selected cancer patients.

**Supplementary Information:**

The online version contains supplementary material available at 10.1186/s13045-022-01289-1.

**To the editor**

International evidence-based clinical practice guidelines (CPGs), which provide recommendations for the best available care options and guide clinical decision-making, have progressively endorsed direct oral anticoagulants (DOACs) as an alternative to monotherapy with low-molecular-weight heparins (LMWHs) for the initial and long-term treatment of cancer-associated thrombosis (CAT) [1–4]. Several new randomized controlled trials (RCTs) have recently reported additional results on the safety and efficacy of DOACs in this setting. Here, we perform an updated study-level meta-analysis of all publicly available results from RCTs comparing DOACs with LMWHs for the treatment of CAT. The literature search and selection process identified 6 RCTs meeting the inclusion criteria [5–10], which were further included in the pooled-analyses (Additional File 1). Together, these trials enrolled a total of 3690 patients with acute CAT (1850 randomized to the DOACs arms and 1840 randomized to the LMWHs arms). Study characteristics are depicted in Table 1. All studies were open label, used a blinded central outcome adjudication design and were estimated to have low risk for performance and detection bias (Additional File 1). During a 3–6 months follow-up under anticoagulant treatment (intention-to-treat population), recurrent venous thromboembolism (VTE) occurred in 99 of 1850 patients receiving DOACs *vs.* 152 of 1840 patients receiving LMWHs. The risk of recurrent VTE was significantly lower with DOACs compared to LMWHs (RR, 0.67; 95%CI, 0.52–0.85; *p* = 0.001; I^2^ = 0%; Fig. 1). With a rate of VTE recurrence of 8.3% in patients receiving LMWHs, the absolute risk reduction with DOACs was 2.7% (95%CI, –4 to –1.2; high certainty of evidence). Major bleeding occurred in 80 of 1850 patients receiving DOACs *vs.* 68 of 1840 patients receiving LMWHs. Although the risk of major bleeding was numerically higher with DOACs, this difference did not reach statistical significance (RR, 1.17, 95%CI, 0.82–1.67; *p* = 0.39; I^2^ = 12%; Fig. 1). With a risk of major bleeding of 3.7% in the LMWHs group, the absolute risk increase with DOACs was 0.6% (95%CI, –0.7 to 2.5; high certainty of evidence). Clinically relevant nonmajor bleeding (CRNMB) occurred more frequently in patients receiving DOACs compared to those receiving LMWHs (RR, 1.66, 95%CI, 1.31–2.09; *p* < 0.0001; I^2^ = 0%, Fig. 1). With a risk of CRNMB of 5.7% in patients receiving LMWHs, the absolute risk increase with DOACs was 3.8% (95% CI, 1.8–6.2). Finally, the rate of all-cause mortality did not differ between the 2 groups (23.3% in the DOACs arms *vs.* 23.5% in the LMWHs arms; RR, 1.02, 95%CI, 0.89–1.16; *p* = 0.80; I^2^ = 13%, Fig. 1). Per Grading of Recommendations Assessment, Development and Evaluation criteria, the quality of evidence was judged to be high for all outcomes.

**Table 1:** Main characteristics of randomized controlled trials included in the pooled analysis

	HOKUSAI-VTE CANCER		SELECT-D		ADAM-VTE		CARAVAGGIO		CASTA-DIVA		CANVAS	
Study design	Non inferiority Randomized, open label, noninferiority trial with blinded central outcome adjudication		Randomized, open-label, pilot trial with blinded central outcome adjudication		Randomized, open label, superiority trial with blinded central outcome adjudication		Randomized, open label, noninferiority trial with blinded central outcome adjudication		Randomized, open label, noninferiority trial with blinded central outcome adjudication		Randomized cohort of an unblinded hybrid comparative effectiveness non-inferiority trial	
Number of randomized patients	1050		406		300		1170		158		671	
Type of patients included	Patients with active cancer and symptomatic or incidental popliteal, femoral or iliac or IVC DVT, symptomatic or incidental PE		Patients with active cancer and symptomatic DVT, symptomatic PE, or incidental PE		Active cancer patients with acute DVT (including upper extremity), PE, splanchnic or cerebral vein thrombosis		Patients with active or recent cancer and acute DVT or PE		Patients with active cancer and acute DVT or PE at high risk of recurrent VTE		Patients with cancer and acute VTE	
Mean Age (years)	64		67		64		67		69		Not reported	
Male sex	52%		53%		48%		49%		49%		Not reported	
Type of cancers included	Colorectal: 15% Lung: 15% Breast: 12% Genitourinary: 13% Gynecologic: 11% Pancreatic or hepatobiliary: 9% Upper gastrointestinal: 5% Hematological malignancies: 11% Other: 10%		Colorectal: 25% Lung: 12% Breast: 10% Genitourinary: 17% Gynecologic: 10% Pancreatic or hepatobiliary: 8% Upper gastrointestinal: 10% Hematological malignancies: 8% Other: 10%		Colorectal: 16% Lung: 17% Breast: 9% Genitourinary: 9% Gynecologic: 10% Pancreatic or hepatobiliary: 16% Upper gastrointestinal: 4% Hematological malignancies: 8% Other: 11%		Colorectal: 20% Lung: 17% Breast: 13% Genitourinary: 9% Gynecologic: 10% Pancreatic or hepatobiliary: 8% Upper gastrointestinal: 5% Hematological malignancies: 7% Other: 11%		Gastro-intestinal: 20% Lung: 18% Breast: 12% Genitourinary: 13% Gynecologic: 8% Hematological malignancies: 8% Other: 21%		Not reported	
Metastatic disease	52.9%		58.0%		64.3%		67.9%		72.8%		Not reported	
Treatment allocation	Intervention (edoxaban)	Control (dalteparin)	Intervention (rivaroxaban)	Control (dalteparin)	Intervention (apixaban)	Control (dalteparin)	Intervention (apixaban)	Control (dalteparin)	Intervention (rivaroxaban)	Control (dalteparin)	Intervention (DOAC)	Control (LMWH)
	Therapeutic dose of LMWH for at least 5 days followed by edoxaban 60 or 30 mg once daily	Dalteparin 200 IU/kg once daily for 1 month followed by 150 IU/kg once daily	Rivaroxaban 15 mg twice daily for 21 days, followed by 20 mg once daily	Dalteparin 200 IU/kg once daily for 1 month followed by 150 IU/kg once daily	Apixaban 10 mg twice daily for 7 days, followed by 5 mg twice daily	Dalteparin 200 IU/kg once daily for 1 month followed by 150 IU/kg once daily	Apixaban 10 mg twice daily for 7 days, followed by 5 mg twice daily	Dalteparin 200 IU/kg once daily for 1 month followed by 150 IU/kg once daily	Rivaroxaban 15 mg twice daily for 21 days, followed by 20 mg once daily	Dalteparin 200 IU/kg once daily for 1 month followed by 150 IU/kg once daily	Any DOAC at the discretion of the treating investigator in accordance with the drug's FDA package insert	Any approved LMWH at the discretion of the treating investigator in accordance with the drug's FDA package insert
Duration of follow-up	12 months		6 months		6 months		6 months		3 months		6 months	
Primary outcome	Composite of recurrent VTE or major bleeding		Recurrent VTE		Major bleeding including fatal bleeding		Efficacy: Recurrent VTE Safety: Major bleeding		Efficacy: Composite of recurrent VTE and worsening of pulmonary vascular or venous obstruction on systematic examinations Safety: Major bleeding		Efficacy: Recurrent VTE Safety: Major bleeding	
Secondary outcomes	Recurrent VTE Major bleeding CRNMB Mortality		Major bleeding CRNMB Mortality		Recurrent VTE CRNMB Mortality		CRNMB Mortality		CRNMB Mortality			
Recurrent VTE	Intervention	Control	Intervention	Control	Intervention	Control	Intervention	Control	Intervention	Control	Intervention	Control
	7.9%	11.3%	4%	11%	0.7%	6.3%	5.6%	7.9%	6.4%	10.1%	6.1%	8.8%
HR (95% CI) for recurrent VTE	0.71 (95% CI 0.48–1.06)		0.43 (95% CI 0.19–0.99)		0.099 (95% CI 0.013–0.780)		0.63 (95% CI 0.37–1.07)		0.75 (95% CI 0.21–2.66)		Not reported	
Major bleeding	Intervention	Control	Intervention	Control	Intervention	Control	Intervention	Control	Intervention	Control	Intervention	Control
	6.9%	4%	6%	4%	0%	1.4%	3.8%	4%	1.4%	3.7%	5.2%	5.6%
HR (95% CI) for Major bleeding	1.77 (95% CI 1.03–3.04)		1.83 (95% CI 0.68–4.96)		Not estimable		0.82 (95% CI 0.40–1.69)		0.36 (95% CI 0.04–3.43)		Not reported	
CRNMB	Intervention	Control	Intervention	Control	Intervention	Control	Intervention	Control	Intervention	Control	Intervention	Control
	14.6%	11.1%	13%	4%	6.2%	4.9%	9%	6%	10.8%	6.1%	5.8%	2.6%
HR (95% CI) for CRNMB	1.38 (95% CI 0.98–1.94)		3.76 (95% CI 1.63–8.69)		–		1.42 (95% CI 0.88–2.30)		–		Not reported	
Mortality	Intervention	Control	Intervention	Control	Intervention	Control	Intervention	Control	Intervention	Control	Intervention	Control
	39.5%	36.6%	23.6%	27.6%	16%	11%	23.4%	26.4%	25.7%	23.8%	21.5%	18.4%
HR (95% CI) for mortality	1.12 (95% CI 0.92–1.37)						0.82 (95% CI 0.62–1.09)		1.05 (95% CI 0.56–1.97)		Not reported	

*CI* confidence interval, *CRNMB* clinically relevant nonmajor bleeding, *DOAC* direct oral anticoagulant, *DVT* deep vein thrombosis, *LMWH* low-molecular-weight heparin, *HR* hazard ratio, *PE* pulmonary embolism, *VTE* venous thromboembolism

**Fig. 1:**
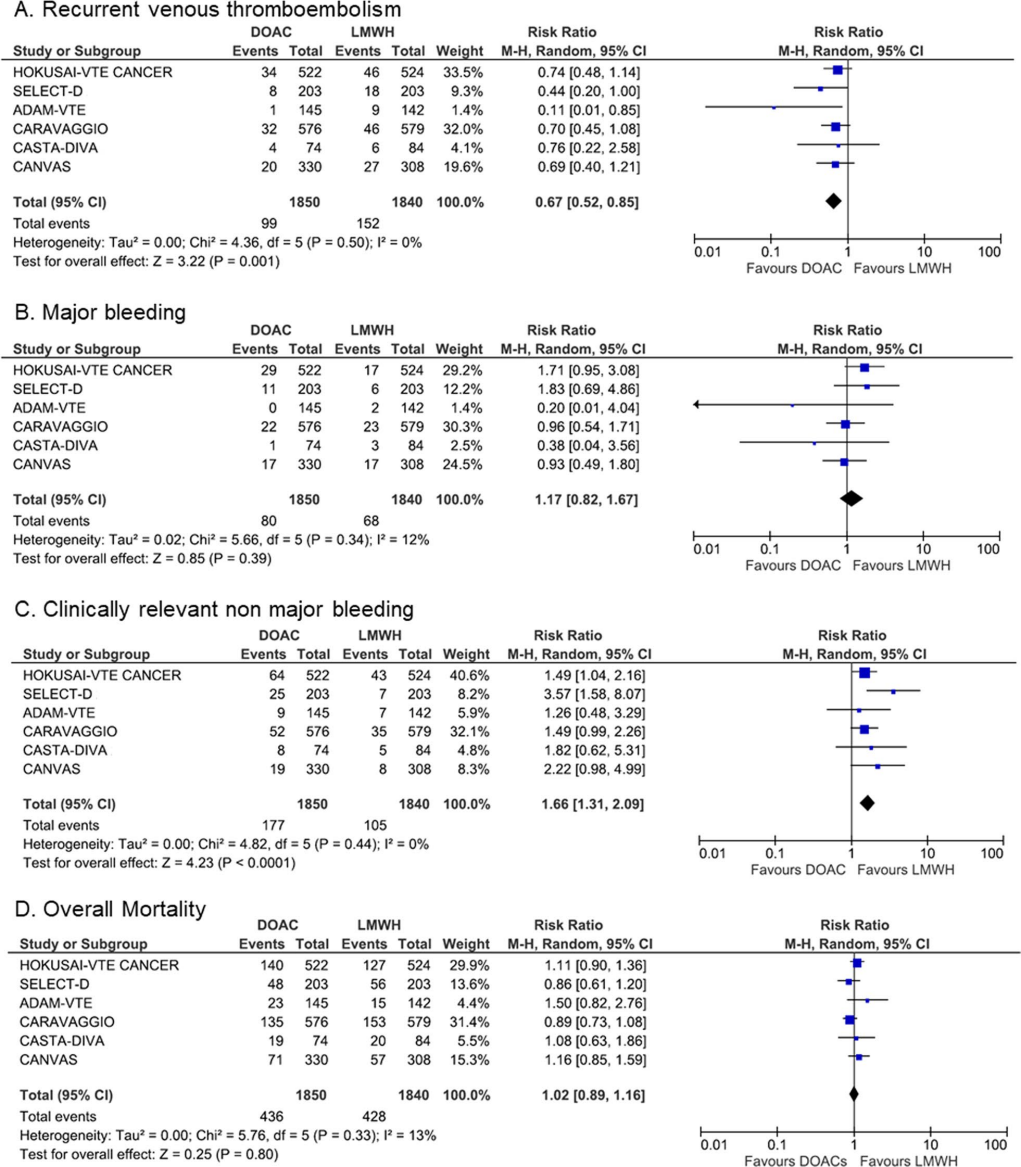
Forest plots of Risk Ratios for Venous Thromboembolism (**A**), Major Bleeding (**B**), Clinically Relevant NonMajor Bleeding (**C**) and Overall Mortality (**D**)

By pooling the results from 6 high quality RCTs, the present study provides more precise estimates of the anticipated treatment effects. Our findings indicate that in cancer patients, DOACs confer a slight reduction in the risk of recurrent VTE. The proportion of patients discontinuing treatment was lower in those randomized to receive a DOAC compared to those randomized to receive a LMWH, which may explain, in part, the superior efficacy of DOACs. The exclusion criteria used in most RCTs (ECOG Performance Status > 2, brain tumors, platelet count < 50–75 G.L^−1^, Cockroft Clairance < 30 ml.min^−1^) may have limited the generalizability of the findings. Importantly, bleeding was more common in patients with gastrointestinal (GI) malignancies receiving edoxaban or rivaroxaban compared with LMWHs [5, 6], while apixaban was not associated with an increased risk of bleeding in these patients [7, 8].

In conclusion, there is growing evidence supporting DOACs as an effective and safe treatment option for VTE in selected cancer patients. Results from the present study increase the level of confidence on available evidence supporting the safety and efficacy of DOACs for the treatment of CAT. LMWHs remain the preferred treatment option in cancer patients at high risk of bleeding, such as GI cancer patients, those who require frequent dose adjustments with chemotherapy-induced thrombocytopenia, those who receive ongoing anticancer therapies with potential drug-drug interactions, as well as those with brain metastases. Dedicated tools, such as the ITAC-CME multi-language web-based mobile application downloadable for free at www.itaccme.com will help to improve the care and quality of life of cancer patients and to further decrease the burden of CAT.

## Supplementary Information

**Additional file 1.** Methods, literature search, summary of finding for pooled analysis.

## Data Availability

The authors confirm that the data supporting the findings of this study are available within the article and its supplementary materials.
