# Supplementary material for: Direct oral anticoagulant versus low molecular weight heparin for the treatment of cancer-associated venous thromboembolism: 2022 updated systematic review and meta-analysis of randomized controlled trials
Source: J Hematol Oncol. 2022 May 21;15:69. doi: 10.1186/s13045-022-01289-1 (PMC9124390; doi:10.1186/s13045-022-01289-1)

Supplementary Data

Methods

The present study was conducted in accordance with the Preferred Reporting Items for Systematic Reviews and Meta-Analyses (PRISMA)[1,2] and registered on PROSPERO (CRD42021266069). We performed a comprehensive literature search using MEDLINE, EMBASE, and Cochrane Central Register of Controlled Trials (CENTRAL) using a predefined search strategy from inception to November 1, 2021 (**Supplementary table 1**). We also hand-searched annual meeting abstracts of American Society of Hematology (ASH), American Society of Clinical Oncology (ASCO), International Society on Thrombosis and Haemostasis (ISTH) and European Society of Cardiology (ESC). Furthermore, unpublished studies were searched on clinicaltrials.gov. We used the Covidence software for systematic reviews (Melbourne, Australia) for records screening. Briefly, 2 reviewers (C.F. and D.F.) independently screened all records identified in the literature search for study eligibility based on title and abstract. Inclusion criteria were: (1) RCTs comparing DOACs *vs*. LMWHs in cancer patients with acute symptomatic or incidental VTE, (2) having a follow-up of at least 3 months, (3) reporting recurrent VTE and/or major bleeding as primary outcome. Any discrepancies in study selection were resolved by consensus and adjudicated by a third author (J.M.C.). In case of duplicate publications, the most recent publication was considered. Data [study procedures (randomization process, treatment allocation, blinding process), intervention and control characteristics (anticoagulant, regimen, duration), patient characteristics (number, mean age, gender, cancer type and staging, anticancer drugs), follow-up (duration, lost to follow-up) and clinical outcomes (recurrent VTE, bleeding, overall mortality)] were independently extracted using dedicated forms. The primary efficacy outcome was recurrent VTE, either symptomatic or incidentally diagnosed, at 3-6 months follow-up, and the primary safety outcome was major bleeding adjudicated according to the criteria of the individual RCT at 3-6 months follow-up. Secondary outcomes included CRNMB, and all-cause mortality. The quality of studies and risk of bias were assessed using the modified Jadad score[3,4] and the Cochrane Risk of Bias Tool for clinical trials[5]. Mantel-Haenszel random-effect (Der Simonian-Laird analysis[6]) was used to estimate

the pooled event-based risk ratio (RR) with 95% confidence interval (CI). Heterogeneity among studies was assessed by the Higgins I^2 statistic, with I^2 <25% representing low degree of heterogeneity. Publication bias was assessed by visual inspection of funnel plots. The anticipated absolute effects expressed as risk difference (and its 95% CI) was calculated based on the baseline risk in the LMWHs group and the relative effect of the intervention (and its 95% CI). All statistical analyses were performed using the Cochrane's Review Manager software (RevMan, version 5.3, Copenhagen, Denmark) and the GRADEpro Guideline Development Tool web application (www.gradepro.org). Institutional review board approval was not required.

Literature search results

The database and manual search identified 376 potentially relevant citations. Seventy-three records were duplicates, 282 were excluded after title and abstract screening, and 21 were assessed for eligibility. Finally, 6 RCTs meeting the inclusion criteria (5 published as full-text articles[7–11], and 1 presented as an abstract at the 2021 ASCO meeting[12]) were further included in this study-level meta-analysis (**Supplementary Figure 1**).

Supplementary references

1 Liberati A, Altman DG, Tetzlaff J, Mulrow C, Gøtzsche PC, Ioannidis JPA, Clarke M, Devereaux PJ, Kleijnen J, Moher D. The PRISMA statement for reporting systematic reviews and meta-analyses of studies that evaluate healthcare interventions: explanation and elaboration. *BMJ* 2009; **339**: b2700.

2 Moher D, Liberati A, Tetzlaff J, Altman DG, PRISMA Group. Preferred reporting items for systematic reviews and meta-analyses: the PRISMA statement. *PLoS Med* 2009; **6**: e1000097.

3 Jadad AR, Moore RA, Carroll D, Jenkinson C, Reynolds DJ, Gavaghan DJ, McQuay HJ. Assessing the quality of reports of randomized clinical trials: is blinding necessary? *Control Clin Trials* 1996; **17**: 1–12.

4 Oremus M, Wolfson C, Perrault A, Demers L, Momoli F, Moride Y. Interrater Reliability of the Modified Jadad Quality Scale for Systematic Reviews of Alzheimer’s Disease Drug Trials. *DEM* Karger Publishers; 2001; **12**: 232–6.

5 Higgins JPT, Altman DG, Gøtzsche PC, Jüni P, Moher D, Oxman AD, Savovic J, Schulz KF, Weeks L, Sterne JAC, Cochrane Bias Methods Group, Cochrane Statistical Methods Group. The Cochrane Collaboration's tool for assessing risk of bias in randomised trials. *BMJ* 2011; **343**: d5928.

6 DerSimonian R, Laird N. Meta-analysis in clinical trials. *Control Clin Trials* 1986; **7**: 177–88.

7 Raskob GE, van Es N, Verhamme P, Carrier M, Di Nisio M, Garcia D, Grosso MA, Kakkar AK, Kovacs MJ, Mercuri MF, Meyer G, Segers A, Shi M, Wang T-F, Yeo E, Zhang G, Zwicker JI, Weitz JI, Büller HR, Hokusai VTE Cancer Investigators. Edoxaban for the Treatment of Cancer-Associated Venous Thromboembolism. *N Engl J Med* 2018; **378**: 615–24.

8 Young AM, Marshall A, Thirlwall J, Chapman O, Lokare A, Hill C, Hale D, Dunn JA, Lyman GH, Hutchinson C, MacCallum P, Kakkar A, Hobbs FDR, Petrou S, Dale J, Poole CJ, Maraveyas A, Levine M. Comparison of an Oral Factor Xa Inhibitor With Low Molecular Weight Heparin in Patients With Cancer With Venous Thromboembolism: Results of a Randomized Trial (SELECT-D). *J Clin Oncol* 2018; **36**: 2017–23.

9 McBane RD, Wysokinski WE, Le-Rademacher JG, Zemla T, Ashrani A, Tafur A, Perepu U, Anderson D, Gundabolu K, Kuzma C, Perez Botero J, Leon Ferre RA, Henkin S, Lenz CJ, Houghton DE, Vishnu P, Loprinzi CL. Apixaban and dalteparin in active malignancy-associated venous thromboembolism: The ADAM VTE trial. *J Thromb Haemost* 2020; **18**: 411–21.

10 Agnelli G, Becattini C, Meyer G, Muñoz A, Huisman MV, Connors JM, Cohen A, Bauersachs R, Brenner B, Torbicki A, Sueiro MR, Lambert C, Gussoni G, Campanini M, Fontanella A, Vescovo G, Verso M, Caravaggio Investigators. Apixaban for the Treatment of Venous Thromboembolism Associated with Cancer. *N Engl J Med* 2020; **382**: 1599–607.

11 Planquette B, Bertoletti L, Charles-Nelson A, Laporte S, Grange C, Mahé I, Pernod G, Elias A, Couturaud F, Falvo N, Sevestre MA, Ray V, Burnod A, Brebion N, Roy P-M, Timar-David M, Aquilanti S, Constans J, Bura-Riviere A, Brisot D, et al. Rivaroxaban versus Dalteparin in Cancer-Associated Thromboembolism: A Randomized Trial. *Chest* 2021; : S0012-3692(21)04079-4.

12 Schrag D, Uno H, Rosovsky RPG, Rutherford C, Sanfilippo KM, Villano JL, Drescher MR, Jayaram NH, Holmes CE, Feldman LE, Zattra O, Cronin C, Basch EM, Weiss A, Connors JM. The comparative effectiveness of direct oral anti-coagulants and low molecular weight heparins for prevention of recurrent venous thromboembolism in cancer: The CANVAS pragmatic randomized trial. *JCO* Wolters Kluwer; 2021; **39**: 12020–12020.

Supplementary Table 1. Literature Search Strategy from inception to November 1, 2021

EMBASE (via Elsevier):

1	'neoplasm'/exp OR 'cancer*':ti,ab OR 'tum*':ti,ab OR 'malign*':ti,ab
2	'venous thromboembolism'/exp OR 'venous thromboembolism':ti,ab OR 'venous thrombosis'/exp OR 'venous thrombosis':ti,ab OR 'deep vein thrombosis'/exp OR 'deep vein thrombosis':ti,ab OR 'pulmonary embolism'/exp OR 'pulmonary embolism':ti,ab
3	'apixaban*':ti,ab OR 'betrixaban*':ti,ab OR 'edoxaban*':ti,ab OR 'rivaroxaban*':ti,ab OR 'dabigatran*':ti,ab
4	'clinical trial'/de OR 'controlled clinical trial'/de OR 'randomized controlled trial'/de OR 'randomiz*':ti,ab
5	#1 AND #2 AND #3 AND #4
6	#5 AND [adult]/lim

MEDLINE (via PubMed):

1	Neoplasms[mesh] OR neoplas*[tiab] OR cancer[tiab] OR malign*[tiab] OR tumor [tiab] OR tumour [tiab]
2	Venous Thromboembolism[Mesh] OR venous thromboem*[tiab] OR Venous Thrombosis[Mesh] OR venous thrombosis[tiab] OR deep vein thrombosis[tiab] OR deep venous thrombosis OR Pulmonary Embolism[Mesh] OR pulmonary embolism[tiab]
3	direct oral anticoagulant OR DOAC OR NOAC OR non-vitamin K antagonist oral anticoagulant OR novel oral anticoagulant OR new oral anticoagulant OR rivaroxaban OR apixaban OR edoxaban OR dabigatran OR betrixaban OR Xa inhibitor
4	randomized controlled trial [pt] OR controlled clinical trial [pt] OR randomized [tiab] OR placebo [tiab] OR clinical trials as topic [mesh: noexp] OR randomly [tiab] OR trial [ti]
5	"Infant"[Mesh] OR "infant"[MeSH Terms:noexp] OR "child"[MeSH Terms] OR "child"[MeSH Terms:noexp] OR infant,newborn[Mesh] OR child,preschool[Mesh]
6	animals [mh] NOT humans [mh]
7	Review[ptyp] OR editorial[ptyp] OR practice guideline[ptyp] OR case reports[ptyp]
8	#5 OR #6 OR #17
9	#1 AND #2 AND #3 AND #4 NOT #8

CENTRAL (via Cochrane):

1	MeSH descriptor: [Neoplasms] explode all trees
2	Neoplasms OR neoplas* OR cancer* OR malign* OR tumor OR tumour
3	MeSH descriptor: [Venous Thromboembolism] explode all trees
4	MeSH descriptor: [Venous Thrombosis] explode all trees
5	venous thromb* OR VTE OR deep vein thrombosis OR deep venous thrombosis
6	MeSH descriptor: [Factor Xa Inhibitors] explode all trees
7	MeSH descriptor: [Dabigatran] explode all trees
8	'apixaban*' OR 'betrixaban*' OR 'edoxaban*' OR 'rivaroxaban*' OR 'dabigatran*' OR betrixaban*' OR DOAC*' OR 'NOAC*' OR 'direct oral anticoagualnt*' OR 'novel oral anticoagulant*' OR 'new oral anticoagulant*'
9	(#1 OR #2) AND (#3 OR #4 OR #5) AND (#6 OR #7 OR #8)

Supplementary Table 2. Summary of findings for pooled analysis of DOAC *vs* LMWH for the treatment of venous thromboembolism in cancer patients.

	№ of participants (Studies)	RR (95%CI)	Observed risk with LMWH	Anticipated absolute effects		Certainty of the Evidence**
				Risk with DOAC (95%CI)	Absolute risk Difference (95%CI)	
**Recurrent VTE**	3690 (6 RCTs)	0.67 (0.52 to 0.85)	8.3%	5.5% (4.3 to 7)	-2.7% (-4 to -1.2)	⊕⊕⊕⊕ High
**Major bleeding**	3690 (6 RCTs	1.17 (0.82 to 1.67)	3.7%	4.3% (3 to 6.2)	0.6% (-0.7 to 2,5)	⊕⊕⊕⊕ High
**CRNMB**	3690 (6 RCTs	1.66 (1.31 to 2.09)	5.7%	9.5% (7.5 to 11.9)	3.8% (1.8 to 6.2)	⊕⊕⊕⊕ High
**All-cause mortality**	3690 (6 RCTs	1.02 (0.89 to 1.16)	23.3%	23.7% (20.7 to 27)	0.5% (-2.6 to 3.7)	⊕⊕⊕⊕ High

Abbreviations: CI, confidence interval; CNRMB, clinically relevant nonmajor bleeding; DOAC, direct oral anticoagulant; LMWH, low-molecular weight heparin; RCT, randomized controlled trials; RR, Risk Ratio; VTE, venous thromboembolism

*The risk in the DOAC group (and its 95% CI) is based on the observed risk in the LMWH group and the relative effect of the intervention (and its 95% CI).

** GRADE Working Group grades of evidence. High certainty: we are very confident that the true effect lies close to that of the estimate of the effect.

Supplementary Figure 1. Literature search strategy

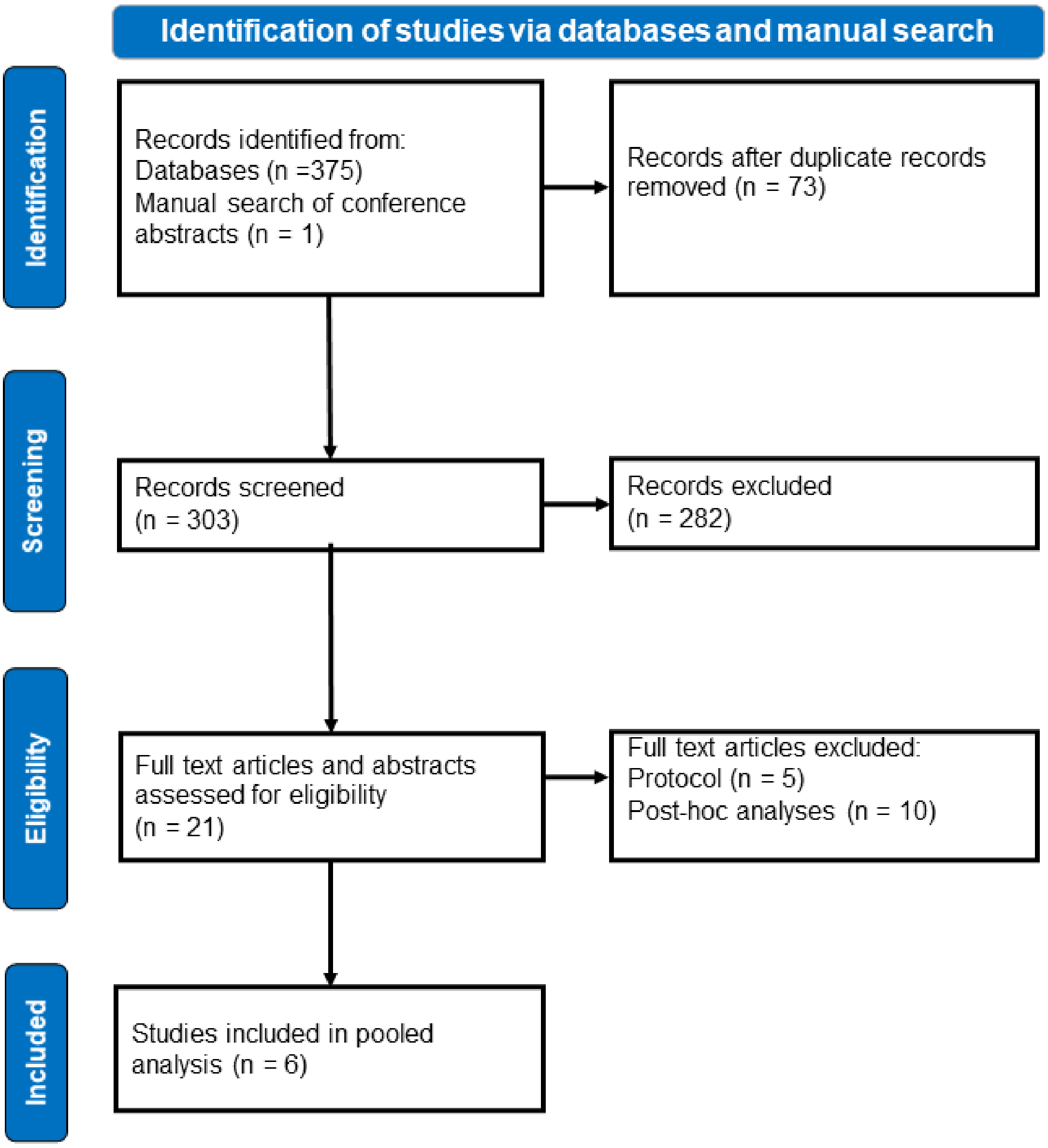

Supplementary Figure 2. Risk of bias assessment

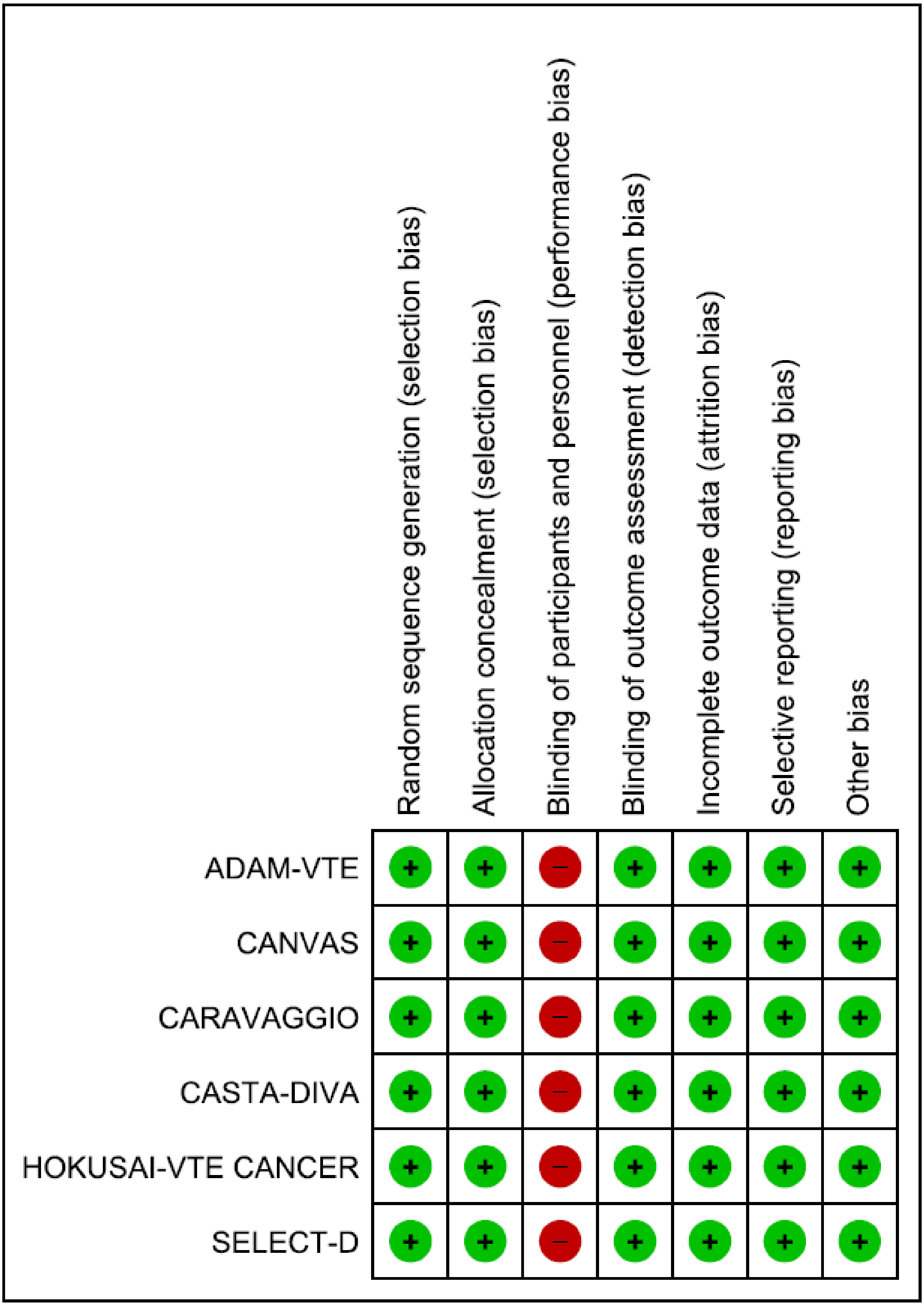

Supplement: Supplementary file 1 — Additional file 1. Methods, literature search, summary of finding for pooled analysis. [file 13045_2022_1289_MOESM1_ESM.pdf]
